# Intramedullary arthrodesis of the knee joint with additional femoral neck screw to prevent periprosthetic fracture of the proximal femur. A case report

**DOI:** 10.3205/iprs000184

**Published:** 2024-02-29

**Authors:** Mohamed Ghanem, Christina Pempe, Andreas Roth

**Affiliations:** 1 Department of Orthopedics, Traumatology and Plastic Surgery, University Hospital Leipzig, Germany; 2 Department of Physical Therapy and Rehabilitation, University Hospital Leipzig, Germany

## Abstract

Arthrodesis of the knee joint has proven effective in the treatment of chronic periprosthetic infections as well as in cases of previous multiple revision surgery after total knee replacement with insufficiency of the extensor apparatus. In this case report, we report on the use of a custom-made intramedullary arthrodesis nail of the knee joint following multiple revisions due to aseptic loosening after total knee replacement. Surgery was performed according to preoperative computerized planning. Microbiological and histological samples obtained intraoperatively showed no evidence of infection. Yet, the patient presented postoperatively with complete loss of active dorsiflexion of the ipsilateral foot. On one-year follow-up, the patient did not complain of any pain. The radiological findings one year after surgery showed no sign of loosening or any other pathological findings. The neurological lesion has completely recovered. The Harris Hip Score HHS improved from 24 (prior to implantation of the arthrodesis) to 75 on one-year follow-up, the Western Ontario and McMaster Universities Osteoarthritis Index WOMAC improved from 86 to 20. The particularity of this case lies in the fact that an additional femoral neck screw was brought in to prevent periprosthetic fracture of the proximal femur. Careful preoperative planning as well as surgical performance were necessary to adjust the rotation of the femoral nail to allow adequate positioning of the femoral neck screw.

Intramedullary arthrodesis of the knee is a suitable management option following multiple revision surgery after total knee replacement with insufficiency of the extensor apparatus. In many cases, an individual therapeutic plan is necessary ranging up to the use of custom-made implants.

## Abstract (de)

Die Arthrodese des Kniegelenks hat sich bei der Behandlung chronischer periprothetischer Infektionen sowie bei vorangegangenen mehrfachen Revisionseingriffen nach Kniegelenkersatz mit Insuffizienz des Streckapparates bewährt. In diesem Fallbericht berichten wir über die Verwendung eines individuell angefertigten intramedullären Arthrodesenagels des Kniegelenks nach mehreren Revisionen aufgrund einer aseptischen Lockerung nach einem Kniegelenkersatz. Die Operation wurde entsprechend der präoperativen computergestützten Planung durchgeführt. Mikrobiologische und histologische Proben, die intraoperativ entnommen wurden, zeigten keine Hinweise auf eine Infektion. Die Patientin stellte sich jedoch postoperativ mit einem vollständigen Verlust der aktiven Extension des ipsilateralen Fußes vor. Bei der Nachuntersuchung nach einem Jahr gab die Patientin keinerlei Schmerzen an. Der radiologische Befund ein Jahr nach der Operation zeigte keine Lockerungszeichen oder andere pathologische Befunde. Die neurologische Läsion hat sich vollständig erholt. Der Harris Hip Score HHS verbesserte sich von 24 (vor der Implantation der Arthrodese) auf 75 nach einem Jahr, der Western Ontario and McMaster Universities Osteoarthritis Index WOMAC verbesserte sich von 86 auf 20. Die Besonderheit dieses Falles liegt darin, dass eine zusätzliche Schenkelhalsschraube eingebracht wurde, um einer periprothetischen Fraktur des proximalen Femurs vorzubeugen. Eine sorgfältige präoperative Planung sowie chirurgische Durchführung waren erforderlich, um die Rotation des Femurnagels anzupassen, so dass eine korrekte Positionierung der Femurhalsschraube möglich war.

Die intramedulläre Arthrodese des Kniegelenks ist eine geeignete Behandlungsoption nach mehrfachen Revisionseingriffen nach totalem Kniegelenkersatz mit Insuffizienz des Streckapparates. In vielen Fällen ist eine individuelle Therapieplanung bis hin zum Einsatz individuell angefertigter Implantate erforderlich.

## Introduction

Arthrodesis of the knee joint has proven effective in the treatment of chronic periprosthetic infections as well as in cases of previous multiple revision surgery after total knee replacement with insufficiency of the extensor apparatus [1], [2], [3], [4], [5], [6]. Intramedullary arthrodesis is of particular importance [1], [2], [3], [4], [5], [6]. In this case report, we report on the use of a custom-made intramedullary arthrodesis nail of the knee joint following multiple revisions due to aseptic loosening after total knee replacement. The particularity of this case lies in the fact that an additional femoral neck screw was brought in to prevent periprosthetic fracture of the proximal femur. Careful preoperative planning as well as surgical performance were necessary to adjust the rotation of the femoral nail to allow adequate positioning of the femoral neck screw.

## Methods

### Case history

**Figure 1:**
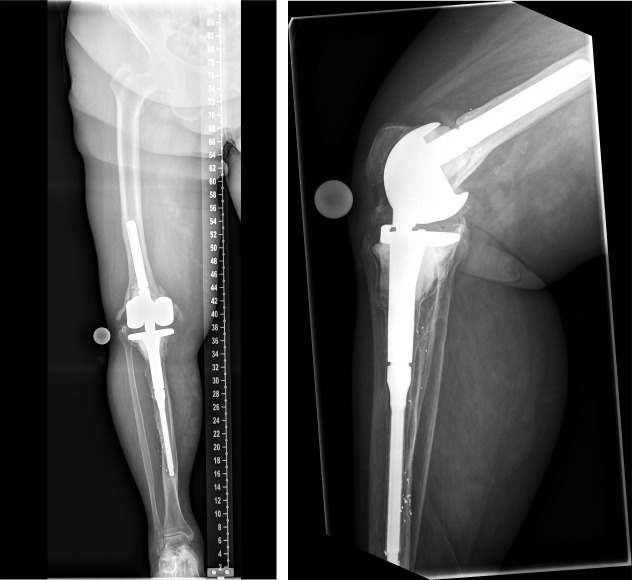
Preoperative X-ray of the right knee joint (in 2016) showing aseptic loosening of the components

**Figure 2:**
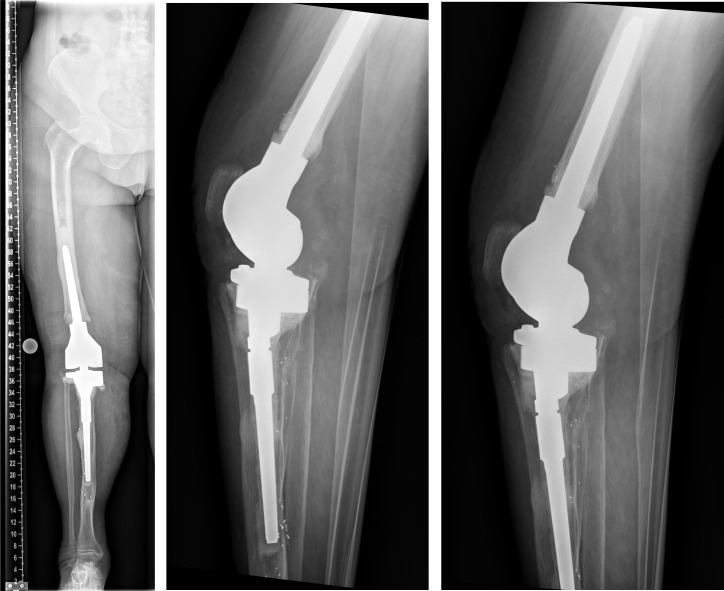
Postoperative X-ray of the right knee joint (in 2016) showing total knee and distal femoral replacement

**Figure 3:**
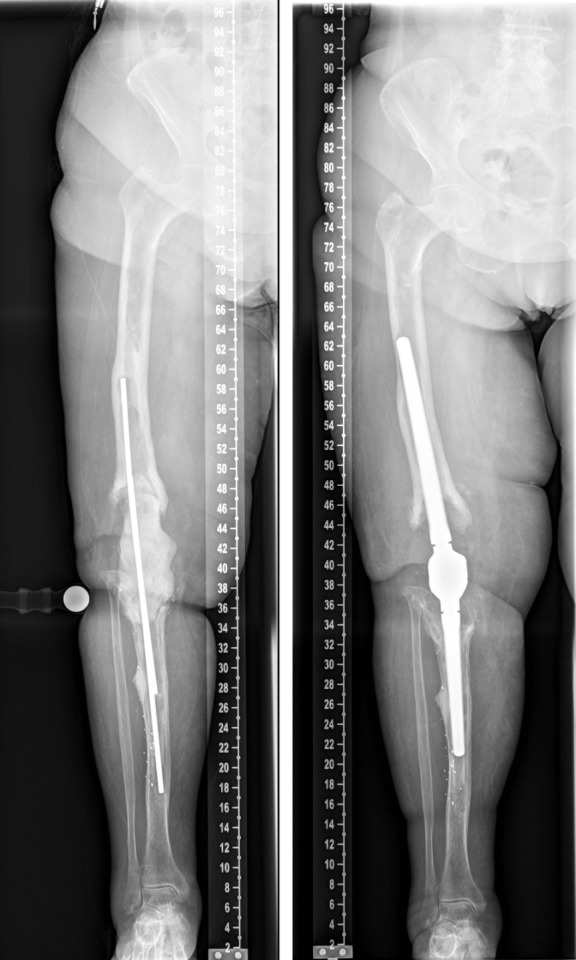
In 2020. Left: Explantation of the components was carried out with implantation of a cement spacer. Right: Intramedullary arthrodesis of the knee

**Figure 4:**
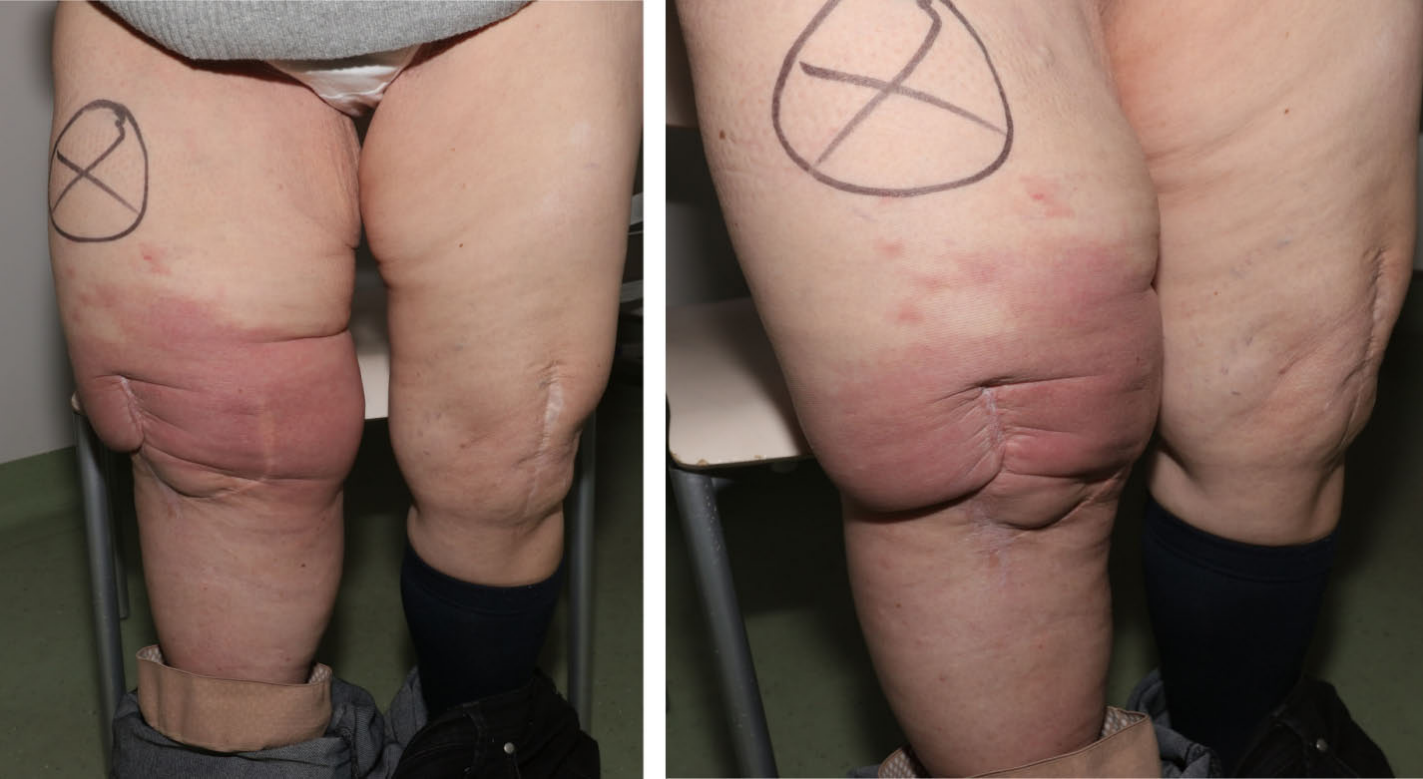
In 2022, local signs of infection

**Figure 5:**
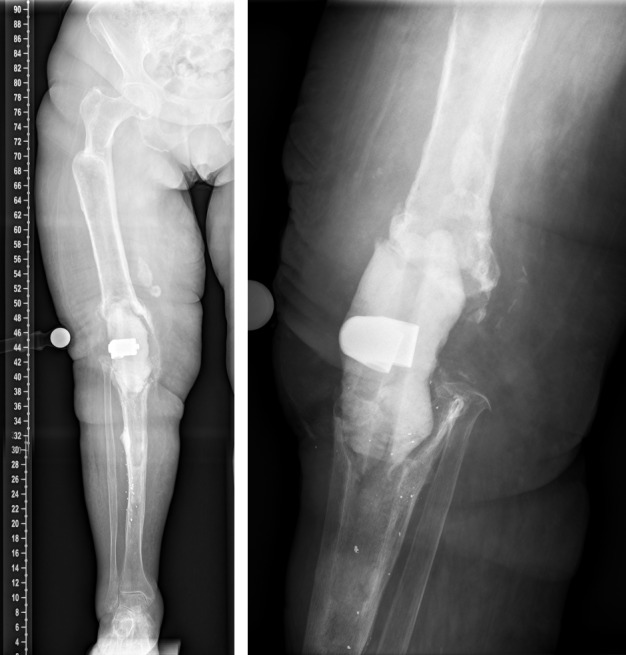
Explantation of the nail was carried out with implantation of cement spacer with intramedullary carbon nails.

This is a report on a female patient aged 80 years at time of last surgery. The patient was first admitted to our center in 2016. Primary right-sided total knee replacement was carried out in 1994, revision surgery due to aseptic loosening was then performed in 2005. In 2016, aseptic loosening of the components was diagnosed again (Figure 1 (Fig. 1)). Revision surgery was carried out with distal femoral replacement (Figure 2 (Fig. 2)). In 2020, the patient presented to us with insufficiency of the extensor apparatus. According to the clinical picture, especially the macroscopic findings during surgery, infection could not be ruled out. Therefore, explantation of the components was carried out with implantation of a cement spacer. The histological and microbiological findings ruled out infection. Therefore, an intramedullary arthrodesis of the knee was carried out during the same hospital stay (Figure 3 (Fig. 3)). In 2022, the patient again complained of pain and we detected loosening of the femoral component. Despite the fact that no microbes were ever isolated in the right knee, we suspected low grade infection, as loosening occurred within less than 2 years accompanied by local signs of infection (Figure 4 (Fig. 4)). Explantation was carried out with implantation of a cement spacer with intramedullary carbon nails (Figure 5 (Fig. 5)). Yet, the histological and microbiological investigations ruled out infection. 

### Planning for surgery

Surgery was indicated for the definitive treatment of the right knee joint after multiple previous operations and insufficiency of the extensor system. Infection has never been proved histologically or microbiologically. In the proximal area of the femur, there was a sclerotic zone with threatening fracture (Figure 5 (Fig. 5)). In order to prevent a fracture in this zone, a femoral implant was specially made into which a femoral neck screw could be inserted. To avoid a via falsa, the sclerotic bone was approached both proximally and distally. According to the instructions of the surgeon, preoperative planning was done and a custom-made implant was manufactured and delivered by PETER BREHM GmbH (Weisendorf, Germany) within 6 weeks (Attachment 1). A special device was introduced to adjust rotation of the femoral nail prior to its implantation in order to adequately place the femoral neck screw (Attachment 1). The surgical procedure was explained to the patient in detail. 

### Surgical procedure

**Figure 6:**
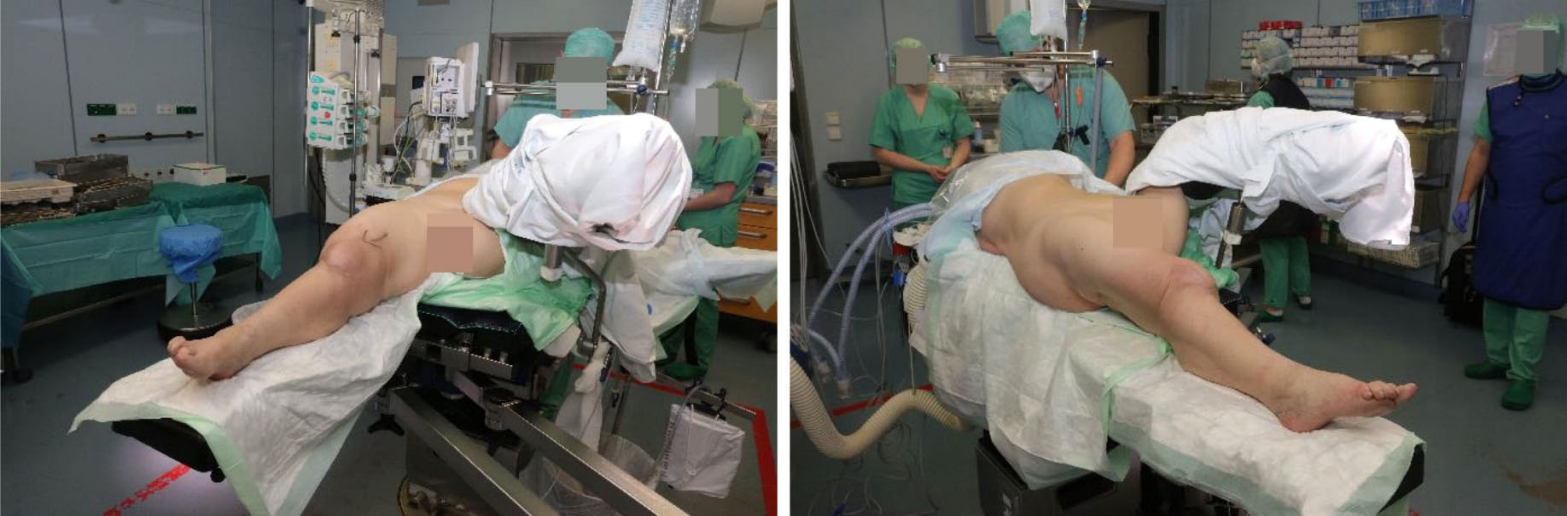
Surgery was performed in supine position allowing intraoperative two-plane imaging of the right lower limb.

**Figure 7:**
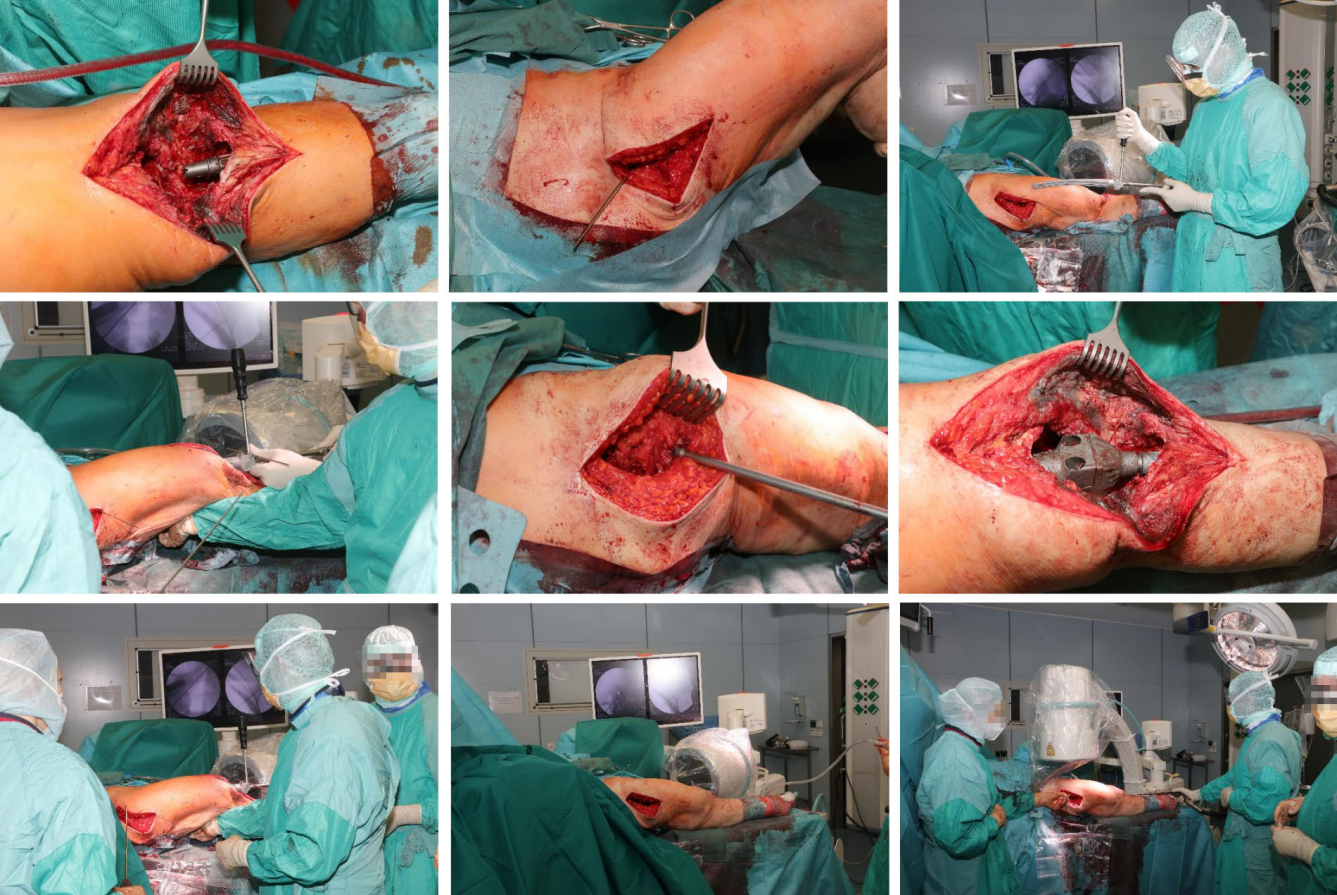
Intraoperative steps (see text) Lateral approach to the thigh, exposure of the femoral shaft and the fracture, exact measurements according to preoperative planning to identify the planned level of resection. Exposure of intramedullary femoral stems, the diaphyseal femur the implants were cemented and additionally secured circularly with screws to the two stem parts after setting the correct rotation. Then, the two parts were connected using the screws provided.

**Figure 8:**
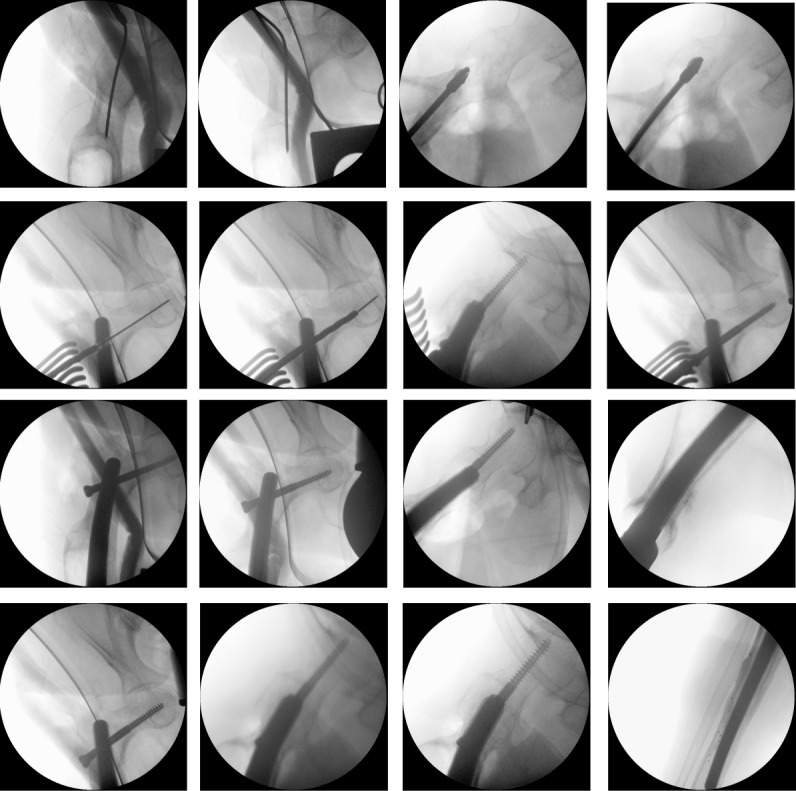
Intraoperative radiographs

Surgery was performed in supine position allowing intraoperative two-plane imaging of the right lower limb (Figure 6 (Fig. 6)). Using the anterior median approach of the knee joint, the cement spacer and the intramedullary carbon nails were removed. There was no macroscopic evidence of infection in the entire surgical area. Nevertheless, swabs were taken from the intra-articular area as well as from the medullary spaces and histopathological specimens from the intra-articular connective tissue. After meticulous debridement and sufficient lavage, the tibial nail was inserted. Afterwards, we performed a lateral approach to the right major trochanter. We introduced an awl under imaging control (Figure 7 (Fig. 7), Figure 8 (Fig. 8)). The sclerosis zone was opened both proximally and distally (Figure 7 (Fig. 7), Figure 8 (Fig. 8)). A Kirschner wire was then inserted into the femoral neck area to mark the entry of the femoral neck screw. The femoral nail was implanted, taking into account the rotation and position of the femoral neck screw. After the Kirschner wire had been removed in the area of the femoral neck, the femoral nail could be driven in further and the femoral neck screw could be properly inserted under imaging control (Figure 7 (Fig. 7), Figure 8 (Fig. 8)).

### Implants used

Custom-made arthrodesis (PETER BREHM GmbH, Weisendorf, Germany), 1 curved athrodesis handle, cement-free KAM-TITAN monoblock femur right size 17/28, 305 mm, 1 straight athrodesis handle, cement-free size 15/200 mm (tibia), knee arthrodesis module right, femoral neck screw 8x 100 cm, MRP shear bolt.

## Result

**Figure 9:**
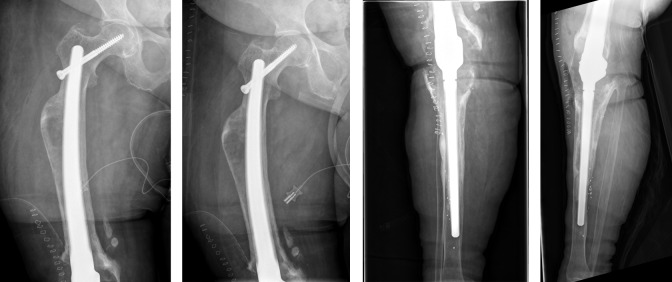
The postoperative radiographs showed correct implant position and a satisfactory surgical result.

The duration of the surgical intervention was four hours and eight minutes. The patient was then mobilized with full weight bearing supervised by physiotherapists at ward level, which she tolerated well. The pain was significantly relieved during the hospital stay. The postoperative radiographs showed correct implant position and a satisfactory surgical result (Figure 9 (Fig. 9)). Microbiological and histological samples obtained intraoperatively showed no evidence of infection. Yet, the patient presented postoperatively with complete loss of active dorsiflexion of the ipsilateral foot. Neurological consultation and investigation confirmed the diagnosis of peroneal lesion. The patient was supplied by orthopedic shoes and orthesis. Further, electrotherapy was carried out.

**Figure 10:**
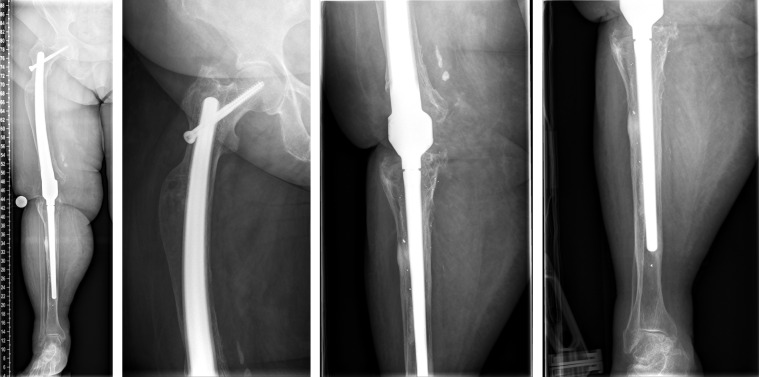
The radiological findings one year after surgery show no sign of loosening or any other pathological findings.

On one-year follow-up, the patient did not complain of any pain. The radiological findings one year after surgery showed no sign of loosening or any other pathological findings (Figure 10 (Fig. 10)). The neurological lesion has completely recovered. The Harris Hip Score HHS improved from 24 (prior to implantation of the arthrodesis) to 75 on one-year follow-up, the Western Ontario and McMaster Universities Osteoarthritis Index WOMAC improved from 86 to 20. The range of motion of the right hip joint one year after surgery was: extension/flexion 0/0/100°, abduction/adduction 30/0/20°, external rotation/internal rotation 30/0/20°.

## Discussion

We report on this case due to the particular feature of the custom-made arthrodesis nail of the knee with the additional femoral neck screw. The challenging part of this surgery was avoiding via falsa or even fracture of the femoral shaft close to the sclerotic zone (Figure 5 (Fig. 5)). Yet, the major technical challenge was introducing the femoral nail in correct rotation to allow adequate positioning of the femoral neck screw. 

Meticulous performance of the surgical procedure was time consuming, hence the rather long duration of surgery. Postoperatively as well as on one-year follow-up, no symptoms or signs of infection were noticed. The peroneal nerve lesion was the only complication encountered after the last surgical procedure and was probably due to intraoperative compression. Complete recovery was noticed after one year. Literature reports revealed that iatrogenic common peroneal nerve lesion could occur in up to 13% of cases [7]. 

Femoral intramedullary nails with antegrade or retrograde insertion options and different locking options have expanded the indications to include diaphyseal and metaphyseal fractures as well as prophylactic surgery to prevent the occurrence of fractures [8], [9], [10]. It must be remembered that retrograde locked nailing of femoral shaft in combination with the insertion of neck screws is a technically demanding but effective procedure [8], [9], [10]. The success rate is high if the surgical procedure is carefully planned and meticulously implemented [8], [9], [10].

The limitation of this study lies in its retrospective design and short follow-up period. However, the patient was 80 years old at the time of surgery and little over 81 at follow-up. Further, most studies on arthrodesis of the knee and on retrograde nailing with subsequent screw fixation of ipsilateral femoral shaft and neck encountered in literature are of retrosprective design.

## Conclusion

Intramedullary arthrodesis of the knee is a suitable management option following multiple revision surgery after total knee replacement with insufficiency of the extensor apparatus. In many cases, an individual therapeutic plan is necessary ranging up to the use of custom-made implants. 

## Notes

### Ethics approval

Approval of the local institutional review board has been given for the study (Ethical Committee at the Medical Faculty, Leipzig University, AZ 020/21-ek) in view of the retrospective nature of the study and all the procedures being performed were part of the routine care.

### Informed consent

The patient has given general consent in the use of their data, including imaging, for analysis and publication. This has been approved by the Ethical Committee. Informed consent was obtained under Ethical approval and consent to participate section.

### Availability of data and material

The datasets used and/or analyzed during the current study are available from the corresponding author on reasonable request.

### Competing interests

The authors declare that they have no competing interests.

### Author’s ORCID

Mohamed Ghanem: 0000-0003-1724-336X

## Supplementary Material

Preoperative planning. © PETER BREHM GmbH
