# Supplementary material for: Intramedullary arthrodesis of the knee joint with additional femoral neck screw to prevent periprosthetic fracture of the proximal femur. A case report
Source: GMS Interdiscip Plast Reconstr Surg DGPW. 2024 Feb 29;13:Doc02. doi: 10.3205/iprs000184 (PMC10963901; doi:10.3205/iprs000184)
Supplement: Preoperative planning. © PETER BREHM GmbH [file IPRS-13-02-s-001.pdf]

Attachment 1

Preoperative planning. Custom-made implants were manufactured and delivered by PETER BREHM GmbH (Weisendorf, Germany). A special device was introduced to adjust rotation of the femoral nail prior to its implantation in order to adequately place the femoral neck screw.

© PETER BREHM GmbH, 2024

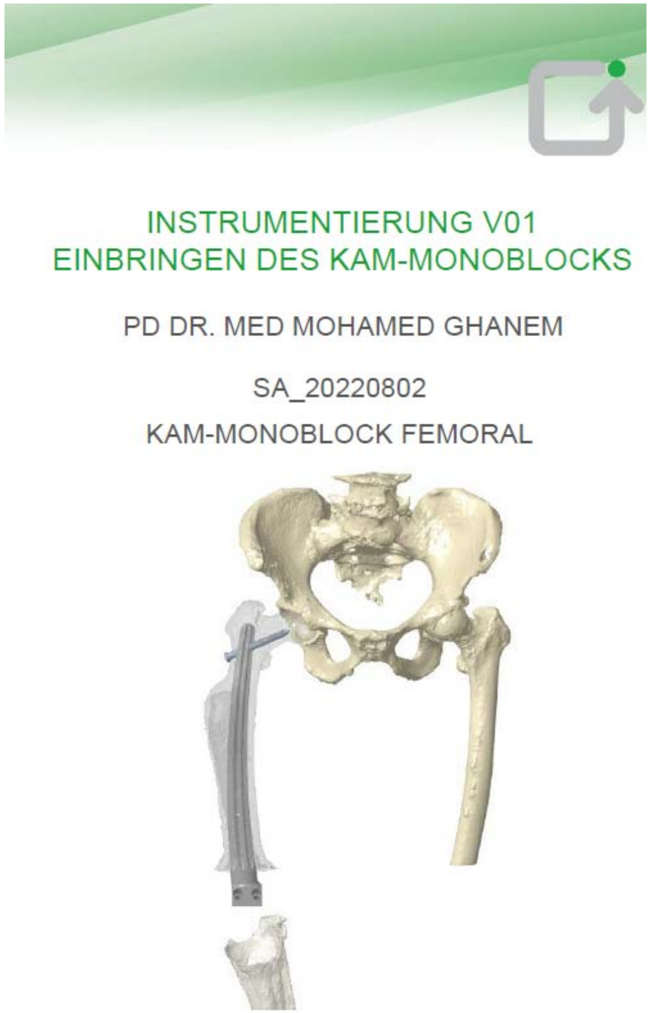

Attachment 1 to: Ghanem M, Pempe C, Roth A. Intramedullary arthrodesis of the knee joint with additional femoral neck screw to prevent periprosthetic fracture of the proximal femur. A case report. GMS Interdiscip Plast Reconstr Surg DGPW. 2024;13:Doc02. DOI: 10.3205/iprs000184

INSTRUMENTIERUNG

VORBEREITUNG FEMUR

PETER BREHM
Die Präzision in Titan für den Menschen

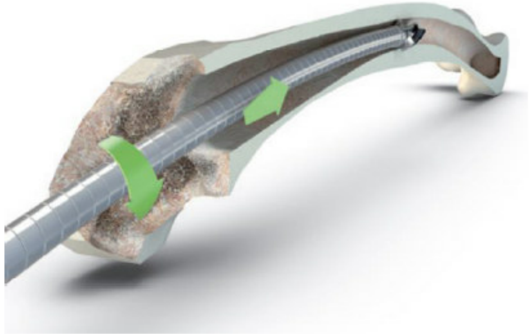

Vorbereitung des Femurs für KAM-Monoblock mit Ø 17/28 mm
unter Berücksichtigung des gewünschten Press-Fits.
Dafür Führungsdraht für flexible Bohrwelle unter
Bildwandlerkontrolle vollständig in den Femur einbringen.
Anschließend Vorbereiten des Markraums unter
Verwendung der flexiblen Bohrwellen

INSTRUMENTIERUNG

EINBRINGEN K-DRAHT

PETER BREHM
Die Präzision in Titan für den Menschen

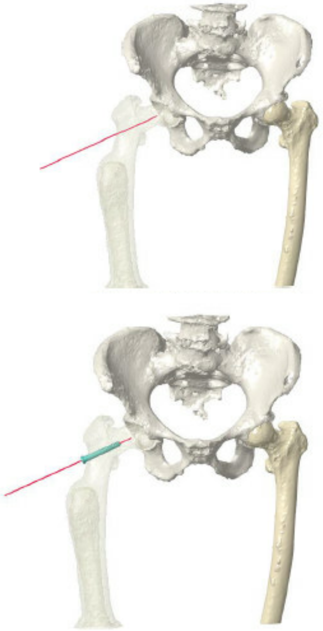

Einbringen eines K-Drahts an Position der Schenkelhalsschraube
mit Hilfe von Bildwandler
Eventuell Einbringen kanulierter Pfahlschraube um Stabilität zu erhöhen

Attachment 1 to: Ghanem M, Pempe C, Roth A. Intramedullary arthrodesis of the knee joint with additional femoral neck screw to prevent periprosthetic fracture of the proximal femur. A case report. GMS Interdiscip Plast Reconstr Surg DGPW. 2024;13:Doc02. DOI: 10.3205/iprs000184

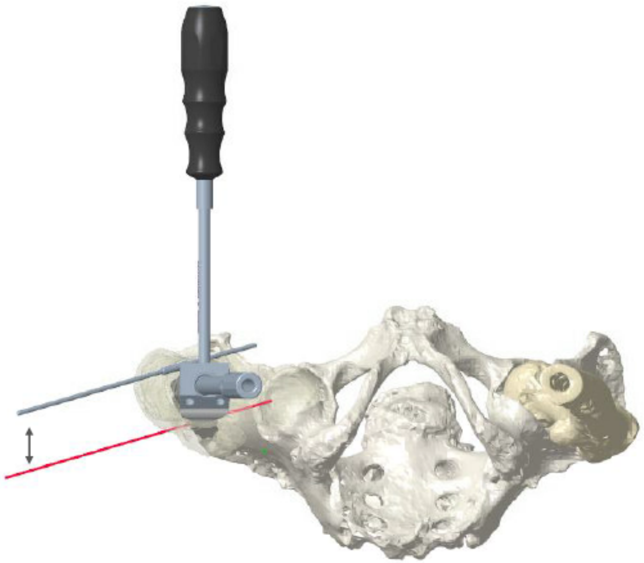

Ausrichten des Zeigers parallel zu K-Draht beim Einschlagen

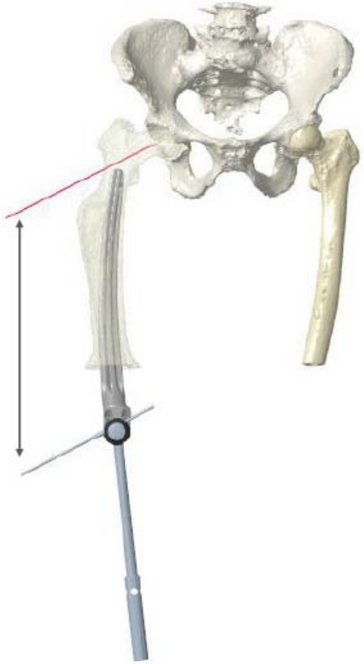

Ausrichten des Zeigers parallel zu K-Draht beim Einschlagen

Attachment 1 to: Ghanem M, Pempe C, Roth A. Intramedullary arthrodesis of the knee joint with additional femoral neck screw to prevent periprosthetic fracture of the proximal femur. A case report. GMS Interdiscip Plast Reconstr Surg DGPW. 2024;13:Doc02. DOI: 10.3205/iprs000184

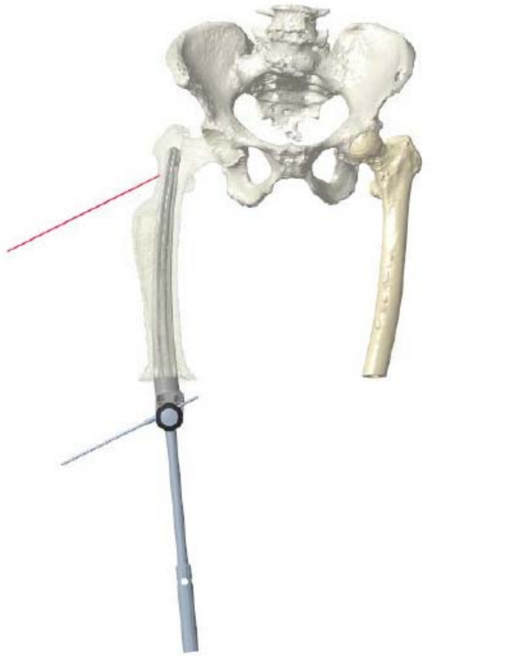

K-Draht soweit herausziehen, dass vollständige Einbringung
des KAM-Monoblocks möglich ist
Anschließend K-Draht wieder in Schenkelhalsbohrung einbringen

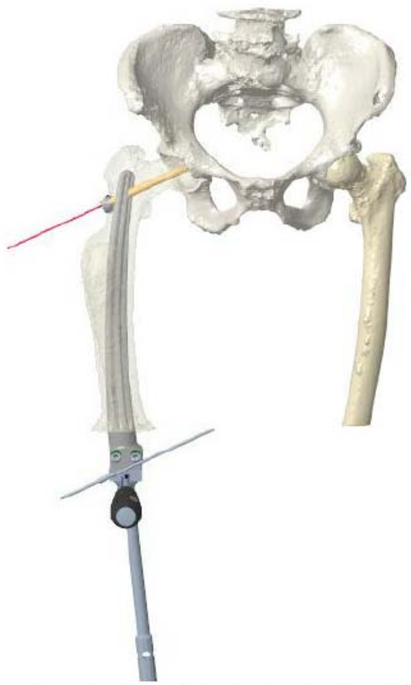

Einschrauben der Schenkelhalsschraube über K-Draht

Attachment 1 to: Ghanem M, Pempe C, Roth A. Intramedullary arthrodesis of the knee joint with additional femoral neck screw to prevent periprosthetic fracture of the proximal femur. A case report. GMS Interdiscip Plast Reconstr Surg DGPW. 2024;13:Doc02. DOI: 10.3205/iprs000184

INSTRUMENTIERUNG

ZUSAMMENBAU KAM-MONOBLOCK MIT GEGENHALTER

PETER BREHM
Die Präzision in Titan
für den Menschen

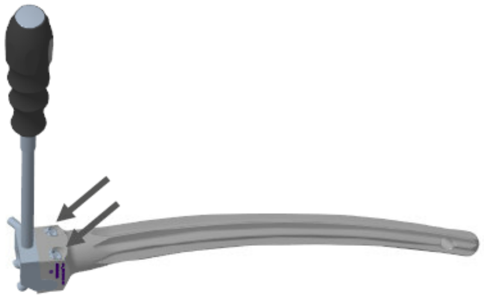

Koppeln des KAM-Monoblocks mit Gegenhalter
(Artnr.: SA_20220802-05) mittels Bolzen
Setzinstrument Prothesenhals einschrauben

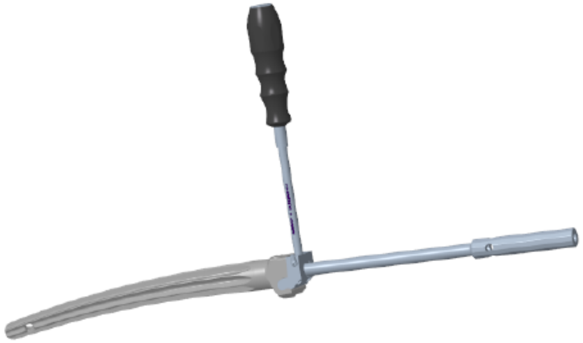

INSTRUMENTIERUNG

EINBRINGEN DES ZEIGERS

PETER BREHM
Die Präzision in Titan
für den Menschen

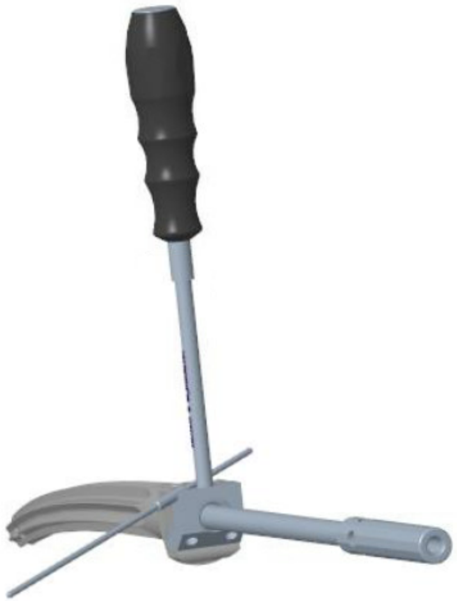

Einbringen des Zeigers Ø 3,2 mm (Artnr. 42700-930-u) in Gegenhalter

Attachment 1 to: Ghanem M, Pempe C, Roth A. Intramedullary arthrodesis of the knee joint with additional femoral neck screw to prevent periprosthetic fracture of the proximal femur. A case report. GMS Interdiscip Plast Reconstr Surg DGPW. 2024;13:Doc02. DOI: 10.3205/iprs000184

VERSORGUNGSVORSCHLAG

ALLGEMEINE INFORMATIONEN

PETER BREHM
Die Präzision in Titan für den Menschen

PATIENTENDATEN	
Patientenname/-ID	[redacted]
Geburtsdatum	[redacted]
Fallnummer	SA_20220802
Erstelldatum CT-Daten	03.08.2022

ECKDATEN / BESCHREIBUNG

Knie-Arthrodesen-Modul femoral rechts
(anatomisch, 6° Valgus, 7° Flexion)

Schaftanteil

- Ø proximal: 17 mm
- Ø distal: 28 mm
- Kurviert
- Verriegelungsmöglichkeit

Schenkelhalsschraube

- Ø 8 x 80 mm (es können verschiedene Längen angeboten werden)

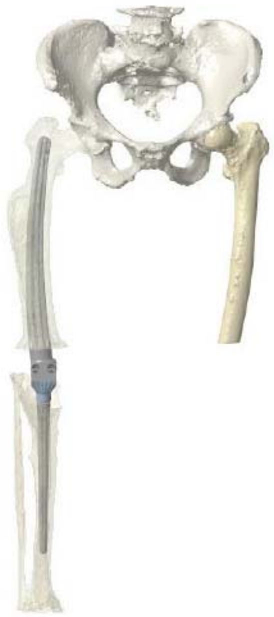

Kontrolle der Beinlänge und –position
Kopplung mit Tibia-Modul und weitere Instrumentierung
gemäß KAM- Instrumentierung

Attachment 1 to: Ghanem M, Pempe C, Roth A. Intramedullary arthrodesis of the knee joint with additional femoral neck screw to prevent periprosthetic fracture of the proximal femur. A case report. GMS Interdiscip Plast Reconstr Surg DGPW. 2024;13:Doc02. DOI: 10.3205/iprs000184
